# Evolving interactions between diazotrophic cyanobacterium and phage mediate nitrogen release and host competitive ability

**DOI:** 10.1098/rsos.160839

**Published:** 2016-12-14

**Authors:** Johannes Cairns, Sebastián Coloma, Kaarina Sivonen, Teppo Hiltunen

**Affiliations:** Department of Food and Environmental Sciences/Microbiology and Biotechnology, University of Helsinki, PO Box 56, 00014 Helsinki, Finland

**Keywords:** cyanobacteria, eco-evolutionary dynamics, *Nodularia* sp., cyanophages, host–parasite interaction, marine nitrogen cycle

## Abstract

Interactions between nitrogen-fixing (i.e. diazotrophic) cyanobacteria and their viruses, cyanophages, can have large-scale ecosystem effects. These effects are mediated by temporal alterations in nutrient availability in aquatic systems owing to the release of nitrogen and carbon sources from cells lysed by phages, as well as by ecologically important changes in the diversity and fitness of cyanobacterial populations that evolve in the presence of phages. However, ecological and evolutionary feedbacks between phages and nitrogen-fixing cyanobacteria are still relative poorly understood. Here, we used an experimental evolution approach to test the effect of interactions between a common filamentous, nitrogen-fixing cyanobacterium (*Nodularia* sp.) and its phage on cellular nitrogen release and host properties. Ecological, community-level effects of phage-mediated nitrogen release were tested with a phytoplankton bioassay. We found that cyanobacterial nitrogen release increased significantly as a result of viral lysis, which was associated with enhanced growth of phytoplankton species in cell-free filtrates compared with phage-resistant host controls in which lysis and subsequent nutrient release did not occur after phage exposure. We also observed an ecologically important change among phage-evolved cyanobacteria with phage-resistant phenotypes, a short-filamentous morphotype with reduced buoyancy compared with the ancestral long-filamentous morphotype. Reduced buoyancy might decrease the ability of these morphotypes to compete for light compared with longer, more buoyant filaments. Together, these findings demonstrate the potential of cyanobacteria–phage interactions to affect ecosystem biogeochemical cycles and planktonic community dynamics.

## 1. Introduction

Cyanobacteria are prominent components of phytoplankton in marine ecosystems, accounting for a significant proportion of global primary production and nitrogen fixation [1–8]. Buoyancy conferred by gas vesicles and the ability to fix atmospheric nitrogen (i.e. diazotrophy) are important factors promoting the competitive dominance of bloom-forming filamentous species in genera such as *Nodularia*, *Aphanizomenon* and *Anabaena* [9,10]. Once blooms have formed, they may be disrupted by physical factors such as intensification of wind [11,12], increase in salinity [13] and decrease in temperature [14], light radiation [15] and the availability of phosphorus [16,17].

Lytic viruses occur in high titres in the upper illuminated layers of the ocean and have been associated with cyanobacterial bloom decay in marine ecosystems across the globe [1,18–22]. However, the more specific role of viruses in bloom termination is relatively poorly understood. In addition to performing photosynthesis, certain cyanobacteria can fulfil their nitrogen demand by nitrogen fixation. Thus, interactions between cyanobacteria and their viruses, cyanophages may play an important role in global biogeochemical cycles [1,23,24]. However, not many studies exist on the eco-evolutionary feedbacks between nitrogen-fixing, or diazotrophic, cyanobacteria and their phages [25]. Fixed nitrogen is released from cyanobacterial blooms, and may be routed to higher trophic levels, through associated heterotrophic bacteria and phytoplankton species [6,7,26,27]. These include members of the nitrogen-limited unicellular picocyanobacterial genera *Synechococcus* and *Prochlorococcus*, which are key primary producers in oceans and display mass occurrence in association with nitrogen-fixing cyanobacteria [2,28,29]. Nitrogenous compounds not only leak out of living cells, primarily as dissolved organic nitrogen and ammonium, but are also released in high concentrations as a result of cellular lysis, such as during bloom decay [26,30]. Because lytic viruses can cause rapid cellular lysis and bloom decay, they can be hypothesized to affect the release of nitrogen from cyanobacterial hosts, altering the flow of nitrogen in planktonic food webs.

Instead of being stable, however, interactions between cyanobacteria and cyanophages can be altered through antagonistic coevolution. Resistance to co-occurring phages has been commonly observed in natural cyanobacterial communities [18,31], suggesting reduction in ecosystem-level effects that result from host lysis. Phage resistance, in turn, has been associated with reduced fitness [32], commonly interpreted as reduced growth rate [33]. However, in a number of studies on picocyanobacteria and their phages, a growth ability cost has not been observed in all resistance mutants [34–37]. Further, it has been proposed that reduced fitness may result from trade-offs between various fitness-related host properties, not only growth ability [33]. Besides affecting host fitness, phages have been found to maintain host diversity at all levels from genomes to populations and communities [38,39]. In addition to the immediate ecological effects resulting from the invasion of a host population by a lytic phage, alterations in host fitness and diversity caused by antagonistic coevolution can thus have wide-ranging ecological and evolutionary consequences.

Here, we used an experimental evolution approach to study the effect of evolving interactions between the filamentous, nitrogen-fixing cyanobacterium *Nodularia* sp. strain AV2 and its lytic phage vB_NpeS-2AV2 (*Siphoviridae*) [25] on cellular nitrogen release and host fitness and diversity. *Nodularia* *spumigena* is among the most common planktonic cyanobacteria and the most important nitrogen-fixers in marine ecosystems such as the Baltic Sea [5,40–42]. We found that rapid evolution in host resistance altered the flow of nitrogen from the cyanobacterium to the environment, with strong community-level effects among other phytoplankton species. Further, we discovered a novel cost of phage resistance, decreased light-competing ability, in association with increased phenotypic diversity in filament morphology and growth ability. These observations shed light on eco-evolutionary dynamics in key species interaction, in our case between nitrogen-fixing cyanobacteria and their phages, which ultimately can have large-scale ecosystems-level effects via altered biogeochemical cycles.

## 2. Material and methods

### 2.1. Test species, culture media and culture conditions

**Figure 1.**
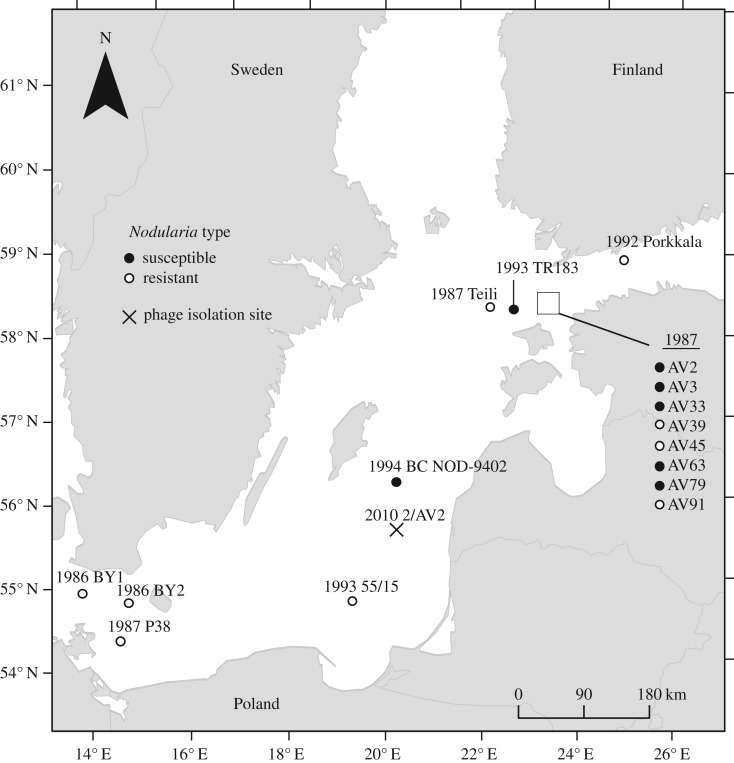
Spatial and temporal occurrence of the *Nodularia* host in the Baltic Sea. (Complete information for strains is provided in the electronic supplementary material, table S2.)

The cyanophage vB_NpeS-2AV2, identified as a member of *Siphoviridae,* was isolated from the surface water in the Baltic proper in June of 2010 after the disappearance of a *Nodularia* bloom [25]. Cyanobacterial host strains of the filamentous, nitrogen-fixing species *Nodularia* sp., isolated from the Baltic Sea, were obtained from the University of Helsinki Culture Collection (HAMBI). The host range of the cyanophage vB_NpeS-2AV2 was tested against 45 *Nodularia* genera isolates from a wide geographical area in the Baltic Sea (figure 1; electronic supplementary material, table S2; for methods, see resistance assay below), isolated between the years 1987 and 1994. When estimating the ecological effects of release of intracellular nitrogen on phytoplankton community, we used 11 phytoplankton strains representing different taxonomic groups, including green algae, diatoms and picocyanobacteria. Details of these strains are provided in the electronic supplementary material, table S1.

In all experiments, we used the cyanobacterial culture medium Z8 with salt and without nitrogen [15,43]. Medium was prepared in glass bottles in type 2 analytical grade water (ELIX® water purification system, Millipak® 40 0.22 µm filter, Merck Millipore, Billerica, MA) and sterilized by autoclaving. All cultures were kept in static conditions in plastic cell culture vials (Sarstedt or VWR) at 25 ± 1°C and a continuous light intensity of 5–8 µmol m^−2^ s^−1^.

### 2.2. Obtaining phenotypes with different evolutionary history

The populations of *Nodularia* sp. strain AV2 studied here were the result of a 22-week-long microcosm experiment consisting of two treatments, host alone (naive population) and host with the phage vB_NpeS-2AV2 (evolved population), with three replicates in both treatments (electronic supplementary material, figure S1; experimental details in [25]). Samples used in this study were collected at the end of the experiment (week 22). From these population-level samples, we isolated approximately 20 clonal strains from each of the three replicates in both treatments (*n* = 118). Strains were isolated and purified by culturing samples in 0.55% agarose plates containing modified Z8 medium. Upon plating, individual filaments were allowed to grow into colonies, and transferred to 20 ml of liquid medium. All isolated clones were tested for phage resistance as described above, and because all clones were cultured in liquid medium for more than 20 generations (more than three months) without the phage, we can safely assume that the resistance trait is heritable.

### 2.3. Identifying potential hosts strains for vB_NpeS-2AV2 phage among the Baltic Sea isolates and testing host resistance

We determined potential host strains for phage vB_NpeS-2AV2 and the phage resistance of experimental isolates with a previously used optical density (OD)-based method [44]. Phage susceptible phenotypes (host strains) and phage-resistant phenotypes were determined as quantitative traits by comparing OD values between cultures with active and inactivated phage, with no difference indicating phage resistance and a significant difference indicating phage susceptibility. In the case of determining host strains among natural isolates from the culture collection (strain details in the electronic supplementary material, table S2), 100 µl of phage stock (approx. 3.6 × 10^7^ pfu ml^−1^) or the same stock inactivated by autoclaving was added to 8 ml containing 2 ml of a dense culture of a *Nodularia* strain and 6 ml of fresh liquid medium in a six-well plate (*n* = 4). After culturing for 7 days, OD at 750 nm was measured (Tecan Infinite M200). Based on the analysis of 45 culture collection isolates, the strain AV2 was chosen as a model for a susceptible *Nodularia* strain in the preceding experimental evolution study [25]. When assessing the resistance of clonal *Nodularia* sp. AV2 isolates obtained from the end of the evolutionary experiment, a volume of 20 µl (containing approx*.* 3.6 × 10^7^ pfu ml^−1^) of ancestral phage or the same stock inactivated by autoclaving was added to 200 µl of clonal host cultures in liquid medium in a 96-well plate (*n* = 4). OD was measured after culturing for 7 and 14–16 days. To test for the possibility of coevolution, we repeated the same assay for all *Nodularia* isolates with 22 week coexistent phage obtained from the same experimental population as the isolate tested.

### 2.4. Measuring evolved changes in *Nodularia* filament length morphology, adhesion and buoyancy

Mean filament length was determined for 34 randomly selected isolates from the naive population and all isolates from the evolved population (*n* = 58). For each isolate, a length-representative sample of 20 filaments was measured using light microscopy. A resistant, short-filamentous type that was detected is hereafter referred as R-short and the ancestral strain-like long type as R-long.

The ability of strains to remain in suspended form in liquid medium was tested for randomly selected naive, R-long and R-short isolates (*n* = 9 for all treatments). A volume of 2 ml from cultures with similar cell densities was added to a 24-well tissue culture plate (Sarstedt), and cultured for 7 days without perturbation. The culture medium and suspended filaments were subsequently removed by a pipette, with special care taken to avoid stirring medium, and the amount of filaments per area in the bottom of the well was counted by light microscopy.

We tested difference in filament buoyancy by taking 1 ml samples of randomly selected long-filamentous and short-filamentous isolates (*n* = 4 for both treatments). The sample was taken from the surface layer of two month old 20 ml cultures with similar overall cell density after the culture had settled for 7 days, as well as after mixing cultures. Difference in sample cell density was then determined by light microscopy.

### 2.5. Measuring growth ability of naive and evolved clones

To examine the effect of phage-mediated evolution on host growth ability, a growth experiment was performed in liquid medium without nitrogen and with limiting concentrations of iron (Fe) or phosphorus (P). Limitation by P and Fe were chosen, because these two are among the key limiting nutrients for cyanobacteria [45,46]. Based on previous studies, concentrations of 2 µM P and 4 µM Fe were selected [17,47]. Chosen concentrations were achieved by adjusting the amount of FeCl_3_ × 6H_2_O in Fe-limiting medium and replacing a part of K_2_HPO_4_ × 3H_2_O with KCl in P-limiting medium. The experiment was performed under both nutrient limitations with the same 27 clones as with the suspension test described above. Prior to beginning the experiment, isolates were washed by centrifugation (7 min at 7000*g*/4°C; repeated once) and nutrient-starved by culturing isolates in low Fe or P conditions for 7 days in order to avoid the transference of excess nutrients into experimental vials. During the experiment, cells were enumerated weekly by light microscopy, and growth rate was calculated as *r* = ln(*N_t _*_+ 1_/*N_t_*)/*t*, where *N_t_* is population size at time *t*.

### 2.6. Release of cellular nitrogen after phage exposure

To test the effect of bacteria–phage interactions on the release of cellular nitrogen and phytoplankton growth ability, populations of *Nodularia* with different evolutionary histories (naive and evolved) were precultured for three weeks in liquid culture medium. Culturing was performed in a volume of 500 ml in a 850 ml tissue culture flask (VWR). After reaching a high cell density (approx. 3.2 × 10^6^ cells ml^−1^), both naive and evolved populations were exposed to the ancestral phage (approx. 4 × 10^7^ pfu ml^−1^) at a high multiplicity of infection of approximately 13. We monitored the following community dynamics by performing host and phage counts daily for 4 days. Host counts were performed by light microscopy, and phage counts were performed with a plaque assay on agarose plates. To acquire cell-free filtrates, cultures at day 4 were centrifuged (7 min at 7000*g*/4°C) and filtered through a 70 µm nylon cell strainer (Falcon®, NY, USA), followed by filtration through 0.22 µm. Total nitrogen in the filtrate was quantified from samples taken at the beginning and end of the experiment by an accredited laboratory (MetropoliLab, Helsinki, Finland) using the standard SFS-EN ISO 11095-1 that follows a previously described method [48].

### 2.7. Effect of *Nodularia* lysate on phytoplankton growth ability

To examine the large-scale effect of bacteria–phage interactions on other members of the phytoplankton community, strains representing green algae, diatoms and picocyanobacteria were cultured in filtrates from naive and evolved population cultures following phage exposure and assumed to contain released cellular nitrogen. The bacteria–phage lysate studies were conducted in nitrogen-free medium (see §2a) with other nutrients at abundant concentrations (i.e. with nitrogen as the only limiting nutrient and nitrogenous compounds from *Nodularia* serving as the only nitrogen source). To allow diatom growth and equalize culturing conditions for all phytoplankton strains, Na_2_SiO_3 _× 9H_2_O was added to a final concentration of 106 mM. In total, 11 phytoplankton strains were studied (electronic supplementary material, table S1). Prior to beginning the experiment, phytoplankton strains were centrifuged and nitrogen-starved for 3 days. We started the growth experiment by adding phytoplankton strains (final cell density of 10^4^ cells ml^−1^) in 40 ml of filtrate in a 75 ml tissue culture flask (Sarstedt). We enumerated cells weekly by using a compound microscope (Zeiss Axioskop 2 plus, Oberkochen, Germany) with a 40× objective and a haemocytometer counting chamber (Improved Neubauer, Marienfeld, Germany). Eukaryotic phytoplankton strains were determined by light microscopy and picocyanobacterial strains (*Synechococcus* and *Synechocystis*) by epifluorescence microscopy. We converted cell numbers to biovolumes using species-specific standardized geometric formulae and size-classes [49].

### 2.8. Statistical analyses

Repeated-measures ANOVA (RMANOVA) was used to analyse host and phage densities and nitrogen concentrations in the nitrogen release test, phytoplankton densities in the bioassay and coevolution of re-infectivity in the phage. One-way ANOVA was used to analyse differences between clonal isolates in filament length, heterocyst density, growth ability, ability to remain in suspended form in medium and formation of oxygenic bubbles. A *t*-test was used to determine the phage resistance of clonal isolates, as well as buoyancy. RMANOVA, one-way ANOVA and *post hoc* analyses were performed with SPSS Statistics v. 22 (IBM SPSS Statistics, Chicago, IL).

## 3. Results

### 3.1 Host resistance evolution and phage coevolution

All isolates from the naive host population (0% resistant, *n* = 60) were susceptible to phage infection, and all isolates from the evolved host population were resistant (100% resistant, *n* = 58). However, within the course of the experiment, re-infectivity detectable with the method used had not evolved in the phage against phage-resistant isolates.

### 3.2 Evolution of new host morphotype

**Figure 2.**
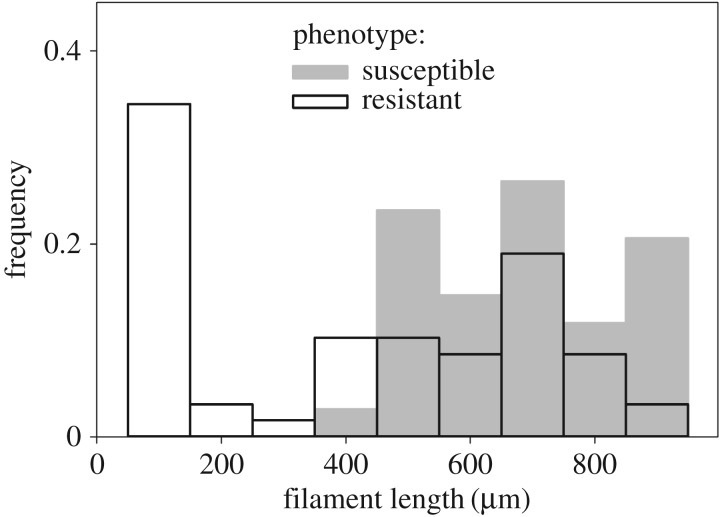
Filament length frequency distribution among susceptible (grey bars, *n* = 34) and resistant phenotype isolates (black bars, *n* = 58). Among resistant isolates, there is a distinct short-filamentous morphotype in addition to an ancestor-like long-filamentous morphotype.

**Figure 3.**
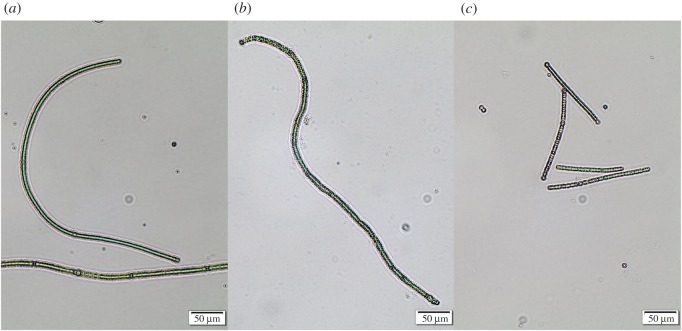
Light micrographs of ancestral and phage-resistant *Nodularia* sp. AV2 isolates. (*a*) Ancestral isolates, (*b*) phage-resistant long-filamentous isolates, and (*c*) phage-resistant short-filamentous isolates.

Based on visual and light-microscopic observations, two morphologically distinct phenotypes were observed among phage-resistant isolates (figures 2 and 3). A novel short-filamentous-resistant phenotype was present in 20.0–57.9% (means: 40.0 ± 19.0%) of isolates from evolved population microcosm replicates, and was not detected among the susceptible phenotype. The short-filamentous phenotype had a highly significantly lower mean filament length compared with both the susceptible phenotype (Tukey's HSD: *p* < 0.001) and the resistant long-filamentous phenotype (Tukey's HSD: *p* < 0.001; ANOVA: *F*_89,2_ = 130, *p* < 0.001). The long-filamentous-resistant phenotype did not differ from the susceptible phenotype (Tukey's HSD: *p* = 0.339).

### 3.3 Differences in growth rates and buoyancy between phenotypes

**Figure 4.**
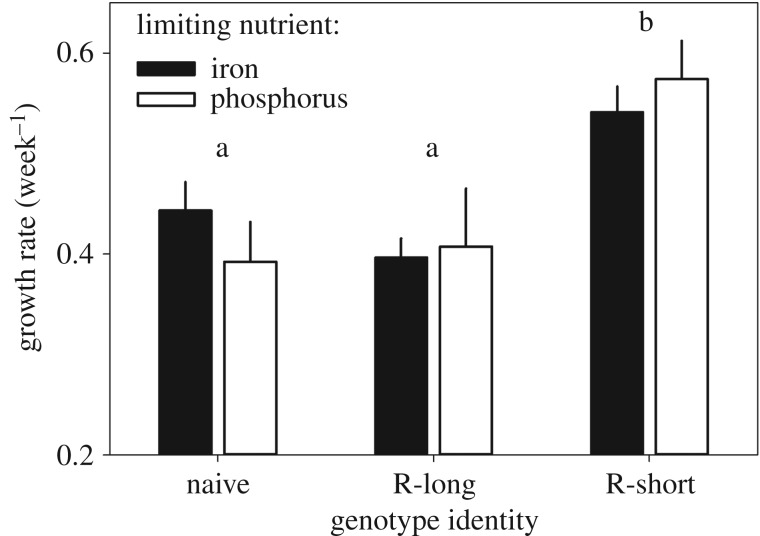
Maximum weekly growth rates (*r*_max_ ± s.e.) of *Nodularia* sp. phenotypes cultured in iron and phosphorus-limited Z8 medium. Different letters indicate significant differences between phenotypes (Tukey's HSD: *p* < 0.05, all differences within phenotypes are non-significant.)

In both iron- and phosphorus-limited conditions, the short-filamentous-resistant phenotype had a higher maximum growth rate compared with both the susceptible phenotype (Tukey's HSD: Fe-lim *p* = 0.026, P-lim *p* = 0.026) and the long-filamentous-resistant phenotype (Tukey's HSD: Fe-lim *p* = 0.002; ANOVA: *F*_21,2_ = 8.75, *p* = 0.002, P-lim *p* = 0.046; ANOVA: *F*_21,2_ = 4.81, *p* = 0.019; figure 4). No difference was observed between the susceptible and resistant long-filamentous phenotypes (Tukey's HSD: Fe-lim *p* = 0.430, P-lim *p* = 0.971).

The ability of the R-short phenotype to stay in suspension in liquid medium was lower (adherence to well plate surface: 1.2 × 10^5^ ± 4.9 × 10^4^ µm mm^−2^) compared with both the susceptible phenotype (2.4 × 10^4^ ± 3.3 × 10^4^ µm mm^−2^; Tukey's HSD: *p* < 0.001) and the R-long phenotype (1.6 × 10^4^ ± 1.5 × 10^4^ µm mm^−2^; Tukey's HSD: *p* < 0.001; ANOVA: *F*_24,2_ = 20.7, *p* < 0.001). The long-filamentous-resistant phenotype did not differ from the susceptible phenotype (Tukey's HSD: *p* = 0.903). Visible oxygen bubble formation in the filament matrix was observed in 97% of susceptible phenotype cultures, 100% of long-filamentous-resistant phenotype cultures and in none of short-filamentous-resistant phenotype cultures, differing from the others (Tukey's HSD: *p* < 0.001; ANOVA: *F*_114,2_ = 904, *p* < 0.001). The long-filamentous-resistant phenotype did not differ from the susceptible phenotype (Tukey's HSD: *p* = 0.651). The density of long-filamentous phenotypes was significantly higher in the surface of the culture compared with the short-filamentous-resistant phenotype (*t*-test: *t*_3.00_ = 4.41, *p* = 0.02), and the densities did not differ in samples collected after mixing cultures (*t*-test: *t*_3.39_ = 1.22, *p* = 0.30).

### 3.4 Release of cellular nitrogen and bioassay with phytoplankton strains

**Figure 5.**
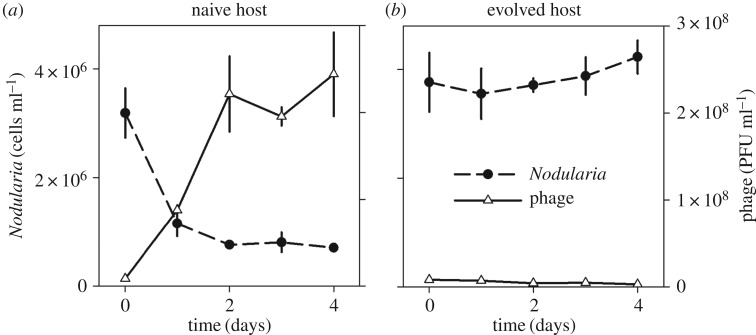
Host (dashed line) and phage (solid line) population dynamics in experimental treatments following phage addition (mean ± s.e.). (*a*) A mass mortality event took place among the naive host population, whereas the evolved host population remained at a high density. (*b*) The phage with naive host increased rapidly, whereas the phage with evolved host remained at a low particle number.

**Figure 6.**
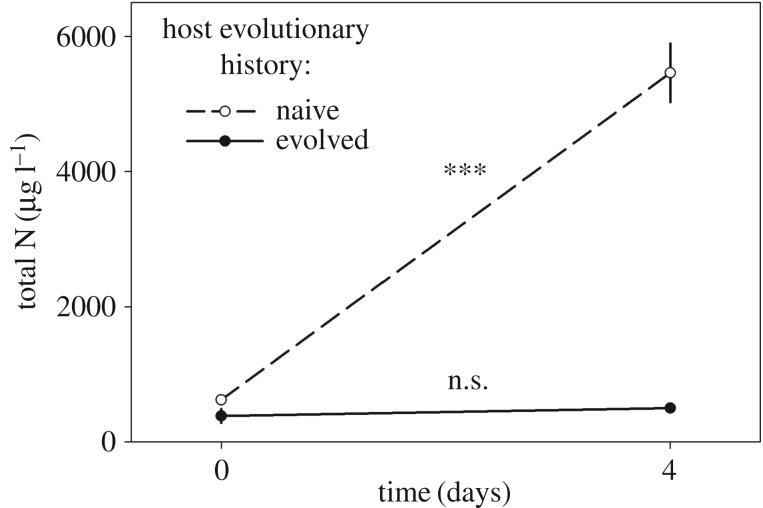
Total nitrogen concentrations in filtrates from experimental treatments at the beginning and end of 4 day-long phage exposure (mean ± s.e.). Total nitrogen increased significantly in the cell-free filtrate from the naive host population (dashed line), whereas remaining at the same level in the filtrate from the evolved host population (solid line).

In the naive population, phage addition resulted in a mass mortality event among the host and substantial increase in phage particle number (figure 5). In the evolved population, phage addition did not result in a change in either host or phage numbers. The treatments differed significantly for both host (*F*_1,20_ = 151, *p* < 0.001) and phage (*F*_1,20_ = 123, *p* < 0.001). Total nitrogen increased in the cell-free filtrate from the naive population, differing significantly from the evolved population in which no increase was detected (*F*_1,4_ = 90.1, *p* < 0.001; figure 6).

**Figure 7.**
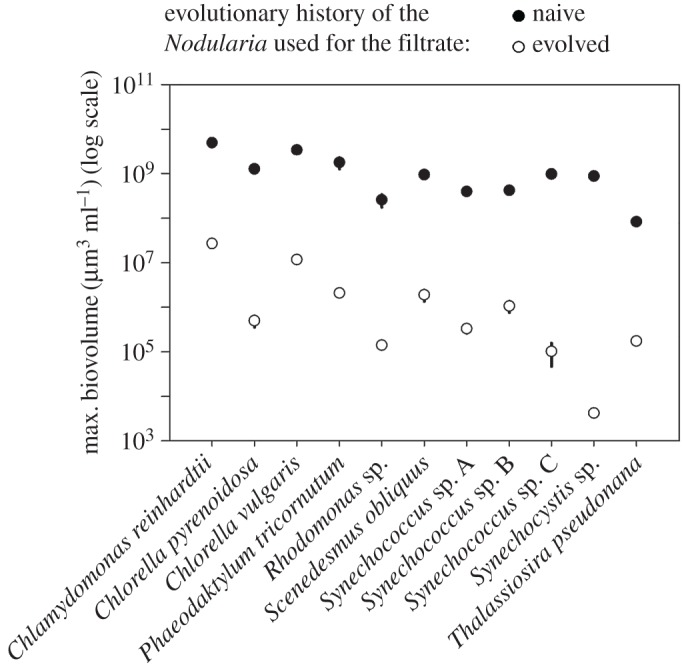
Maximum phytoplankton biovolume sustained by experimental filtrates (mean ± s.e.). The biovolumes of phytoplankton strains cultured in the filtrate from the naive population (black dots) are 1.9 × 10^2^–2.1 × 10^5^ higher (*Chlamydomonas reinhardtii* UTEX 89 and *Synechocystis* sp. UHCC 0318, respectively) compared with the evolved population (*white dots*). (Details of the phytoplankton species used can be found in the electronic supplementary material, table S1.)

All 11 studied phytoplankton strains grew significantly better in the filtrate from the naive population compared with the evolved population (RMANOVA: *p* < 0.001; figure 7).

## 4. Discussion

We found that high levels of cellular nitrogen were released from the nitrogen-fixing cyanobacterium *Nodularia* sp. strain AV2 upon viral lysis. This process can have ecosystem scale effects, because nitrogen-fixing cyanobacteria can reach a high biomass and thus be an important source of nitrogen in aquatic environments. In the case of *Nodularia* and phage vB_NpeS-2/AV2, it also seems that susceptible host types have a rather wide spatio-temporal distribution (figure 1) in the Baltic Sea, highlighting its potential importance concerning the nitrogen cycle. In general, in the Baltic Sea, the filamentous cyanobacteria *Nodularia spumigena*, *Aphanizomenon* spp. and *Anabaena* spp. are considered to be the principal nitrogen-fixing organisms [2,5]. *Nodularia* *spumigena*, in particular, can play a substantial role in the total carbon flux even at low abundance [5]. By causing host lysis and, subsequently, release of cellular nitrogen from the host, cyanophages infecting nitrogen-fixing cyanobacteria can therefore be key players in planktonic communities, even though viral biomass is very small compared with other microbes. In this sense, phages that lyse nitrogen-fixing cyanobacteria can be considered to be keystone species in aquatic ecosystems [50]. This reasoning is in line with previous estimates concerning the significance of the viral loop in rerouting organic matter in marine ecosystems [1,23,24,51].

However, the importance of the link between cyanobacteria–phage interactions and the nitrogen cycle depends on the evolutionary background of the host. Evolution of host resistance, a common occurrence in cyanobacterial populations in nature and the laboratory alike [18,20,21,23,25,31,33,34,36,37,52] can reduce the importance of this link. This is evidenced by our observation that cellular nitrogen release did not occur with phage resistance phenotypes of *Nodularia* sp. Furthermore, in the Baltic Sea, *Nodularia* sp. AV strains that were isolated from the same bloom within a 8 day time window, showed variability in phage susceptibility, suggesting that both susceptible and resistant phenotypes can coexist in a single bloom (figure 1). This indicates the possibility for temporal phenotype dynamics in natural blooms. Antagonistic coevolution between nitrogen-fixing cyanobacteria and their phages may therefore cause spatio-temporal changes in the availability of dissolved organic nitrogen, contributing to a dynamic nutrient landscape in planktonic ecosystems. In addition to causing nitrogen release via lysis of sensitive host cells, cyanophages might also alter the nitrogen metabolism of live cyanobacteria by expression of specific proteins, as has been shown for carbon metabolism [53].

Evolution in key species interaction can have large, community-level effects. In experimental evolution studies, dynamics have been traditionally studied with simple one resource one consumer communities, such as bacteria–phage [54–60], bacteria–protozoan [61–63] or algae–rotifer [64–66] systems. However, experimental evidence on eco-evolutionary community dynamics is also needed in more complex food webs. Nevertheless, to date, a relatively low number of studies have addressed the effects of evolution on ecological dynamics in multispecies communities [67]. Here, we present indirect evidence of the effect of evolution in one key interaction on biologically relevant community dynamics. We found that strains representing common phytoplankton groups, green algae, diatoms and picocyanobacteria, grew considerably better in cell-free filtrates from phage-exposed susceptible host cultures compared with resistant host cultures. These results indicate that eco-evolutionary dynamics in a focal host–parasite interaction can resonate into wider community dynamics via causing fluctuations in resource release. More specifically, these observations also support the hypothesis that passing of fixed nitrogen from cyanobacterial blooms to nitrogen-limited picocyanobacteria may explain their mass occurrence in association with nitrogen-fixing cyanobacteria [2,28]. This specific interaction is especially significant since picocyanobacteria are among the most important photosynthetic organisms in marine ecosystems.

We also found that evolution of phage resistance resulted in a novel short-filamentous *Nodularia* sp. morphotype with decreased buoyancy and increased growth ability compared with other resistant and susceptible isolates. This observation has several potential ecological and evolutionary consequences. First, this lends support from a novel filamentous cyanobacteria–phage system to the hypothesis that phages act to maintain bacterial diversity [38,39]. Second, we propose that decreased buoyancy is a new type of fitness cost caused by phage resistance, because buoyancy can be considered to be critical to the survival of cyanobacteria. Because photosynthesis is their primary mode of energy metabolism, decreased buoyancy may significantly lower cyanobacterial light-competing ability. By allowing upward movement in the vertical light gradient of the water column, bloom-formation and overshadowing of competing phototrophs [9,68], the light-competing ability conferred by buoyancy can be considered to be an essential component in both the interspecies and intraspecies competitive ability of cyanobacteria. In this context, a fitness trade-off may be considered to occur between phage resistance and competition for nutrients and light, with reduced overall fitness in a subset of the resistant population. Development of large phenotypic differences may also lead to expansion into new ecological niches, such as from planktonic to benthic zones as is imaginable in the case of reduced buoyancy, facilitating genetic divergence between morphotypes through adaptive radiation [69]. This can be important since the genus *Nodularia* includes not only planktonic, but also benthic species, *N*. *sphaerocarpa* and *N*. *harveyana* [70–72], viruses can be speculated to play a role in niche diversification processes. A low number of *Nodularia* species supported by molecular studies is contrasted by a much higher number of traditional taxonomical species assignments based on morphological diversity [73,74]. Our observations give rise to the question of whether the high observed morphological diversity can, in part, be explained by phage-mediated evolution, potentially promoting intraspecies adaptive radiation. These findings may have implications concerning the evolutionary history and diversification of filamentous nitrogen-fixing cyanobacteria.

The observations in this study demonstrate the potential of interactions between diazotrophic cyanobacteria and their phages to have large-scale ecosystem effects. Future directions include focusing on more specific aspects of the system, such as the molecular basis of phage resistance and differential filament morphology, and the potential fitness trade-offs associated with each identified phenotype, to provide a more explicit understanding of the mechanistic basis of these effects.

## Supplementary Material

Data from the long term experiment and phytoplankton table Nodularia table

## Data accessibility

Data available from the Dryad Digital Repository: http://dx.doi.org/10.5061/dryad.nd58h [75].
