# Supplementary material for: Evolving interactions between diazotrophic cyanobacterium and phage mediate nitrogen release and host competitive ability
Source: R Soc Open Sci. 2016 Dec 14;3(12):160839. doi: 10.1098/rsos.160839 (PMC5210698; doi:10.1098/rsos.160839)
Supplement: Data from the long term experiment and phytoplankton table Nodularia table [file rsos160839supp1.docx]

Supporting Information for

**Evolving interactions between diazotrophic cyanobacterium and phage mediate nitrogen release and host competitive ability**

Johannes Cairns, Sebastián Coloma, Kaarina Sivonen and Teppo Hiltunen

**This PDF file includes:**

**Supporting figures**

**Fig. S1** Evolutionary history of experimental populations.

**Supporting tables**

**Table S1** Phytoplankton strains used in the study.

**Table S2** *Nodularia* strains from the Baltic Sea used for screening of host range.

**References**

**Fig. S1.** Evolutionary history of experimental populations (mean ± S.E.). Samples were collected from the end of the experiment (week 22). **(a)** The phage sensitive genotype dominated population consists of the host *Nodularia* sp.AV2 (*solid line*) that has evolved alone. **(b)** The phage resistant genotype dominated population consists of the host that has evolved together with the phage vB_NpeS-2/AV2 (*dashed line*). Red circle indicates the time point of isolation of the clones used in this study. Data from Coloma et al. unpublished manuscript (2016).

**Table S1.** Phytoplankton strains used in the study.

| Strain identity | Phylum | Origin | Reference |
| --- | --- | --- | --- |
| *Chlamydomonas reindardtii* UTEX 89 | Chlorophyta | MA, USA | 1 |
| *Chlorella pyrenoidosa* TV 216 | Chlorophyta | Baltic Sea | 2 |
| *Chlorella vulgaris* UTEX 26 | Chlorophyta | CZ | 1,3 |
| *Phaeodaktylum tricornutum* TV 335 | Bacillariophyta | Baltic Sea | 2 |
| *Rhodomonas* sp. Crypto07-B1 | Cryptophyta | Baltic Sea | 4 |
| *Scenedesmus obliquus* | Chlorophyta | Unknown | 5 |
| *Synechococcus* sp. CCY 0417 | Cyanobacteria | Baltic Sea | 6 |
| *Synechococcus* sp. CCY 0435 | Cyanobacteria | Baltic Sea | 6 |
| *Synechococcus* sp. TV65 | Cyanobacteria | Baltic Sea | 2 |
| *Synechocystis* sp. UHCC 0318 | Cyanobacteria | Baltic Sea | 7 |
| *Thalassiosira pseudonana* TV5 | Bacillariophyta | Baltic Sea | 2 |

*1* UTEX = The Culture Collection of Algae at the University of Texas at Austin, *2* Tvärminne Zoological Station of the University of Helsinki (described in Hällfors & Hällfors 1992), *3* Yoshida et al. 2004, *4* courtesy of Anke Kremp, Tvärminne Zoological Station, *5* Max-Planck Institute für Limnologie, *6* Haverkamp et al. 2009 (CYY = Culture Collection Yerseke), *7* University of Helsinki Culture Collection.

**Table S2**. *Nodularia* strains isolated from the Baltic Sea used for screening of host range.

| Strain | Year of isolation | Host^a^ | References |
| --- | --- | --- | --- |
| 0208Porkkala | 1992 | ○ | UHCC |
| 54/13 ( = EIB) | 1993 | ○ | 1 |
| 55/15 ( = EIA) | 1987 | ○ | 1,9 |
| AN13a | 1994 | ● | 2 |
| AN13c | 1994 | ● | 2 |
| AV2 | 1987 | ● | 1,3,4 |
| AV3 | 1987 | ● | 1,9 |
| AV33 | 1987 | ● | 1,9 |
| AV39 | 1987 | ○ | 1 |
| AV45 | 1987 | ○ | 1 |
| AV63 | 1987 | ● | 1 |
| AV79 | 1987 | ● | 1 |
| AV91 | 1987 | ○ | 1 |
| BC Nod-9402 | 1994 | ● | 5 |
| BY1 | 1986 | ○ | 6,7,9 |
| BY2 | 1986 | ○ | 6 |
| CH301 | ̶̶ ^b^ | ○ | CH |
| CH307 | 1987 | ○ | 8 |
| CH311 | 1987 | ○ | 8,13 |
| FL2 | 1994 | ○ | UHCC |
| GR6 | 1992 | ○ | UHCC |
| GR7b | 1992 | ○ | UHCC |
| GR8a | 1992 | ○ | 9 |
| GR8b | 1992 | ○ | 8-10 |
| GR9c | 1992 | ○ | 2 |
| GR9d | 1992 | ○ | UHCC |
| HV-36 | 1991 | ○ | 2 |
| LL12 | 1993 | ○ | 11 |
| P38 ( = EIB) | 1987 | ○ | 1,2,9 |
| PCC 9350 ( = axenic AV2) | 1987 | ● | 4,12 |
| Sr5a | 1991 | ● | 2 |
| SR5i | 1991 | ● | 2 |
| Teili | 1987 | ○ | 1,9 |
| TR183 | 1993 | ● | 2,9 |
| TR193 | 1993 | ○ | UHCC |
| TR291a | 1994 | ○ | UHCC |
| TRO12b | 1994 | ○ | UHCC |
| TRO12d | 1994 | ○ | UHCC |
| TRO31a | 1994 | ○ | 2 |
| TRO31b | 1994 | ○ | UHCC |
| WP2a | 1994 | ○ | UHCC |
| WP2b | 1994 | ○ | UHCC |
| WP2c | 1994 | ○ | 2 |
| WP2d | 1994 | ○ | 2 |
| WP2f | 1994 | ○ | 2 |

^a^ The sensitive host strains were lysed (●) and the resistant strains where not affected (○). ^b^ Not known (–). Culture collections: PCC, Pasteur Culture Collection of Cyanobacteria; CH, Hübel, M. Hübel of E.-M.-Arndt University Greifswald, Biological Station Hiddensee, Kloster Germany; UHCC, University of Helsinki Cyanobacterial Collection HAMBI/UHCC. Numbers indicate the following references: *1* Sivonen *et al.* 1989a, *2* Allahverdiyeva *et al.* 2010, *3* Koskenniemi *et al.* 2007, *4* Martin *et al.* 1990, *5* Hayes *et al.* 1997, *6* Sivonen *et al.* 1989b, *7* Moffit *et al.* 2001, *8* Fewer *et al.* 2013, *9* Lehtimäki *et al.* 2000, *10* Laamanen *et al.* 2001, *11* Kononen *et al.* 1993, *12* Iteman *et al.* 2002, *13* Lyra *et al.* 2005.

**References**

**Allahverdiyeva Y, Leino H, Saari L, Fewer D, Shunmugam S, Sivonen K, Aro E-M. 2010.** Screening for biohydrogen production by cyanobacteria isolated from the Baltic Sea and Finnish lakes. Int. J. Hydrogen Energ. 35:1117–1127.

**Coloma SE, Dienstbier A, Roine E, Bamford D, Sivonen K, Hiltunen T.** Newly isolated *Nodularia* phage influences cyanobacterial community dynamics. Unpublished manuscript.

**Fewer DP, Jokela J, Paukku E, Österholm J, Wahlsten M, Permi P, Aitio O, Rouhiainen L, Gomez-Saez GV, Sivonen K. 2013.** New structural variants of aeruginosin produced by the toxic bloom forming cyanobacterium *Nodularia spumigena*. PLoS One 8:e73618.

**Haverkamp THA, Schouten D, Doeleman M, Wollenzien U, Huisman J & Stal LJ. 2009.** Colorful microdiversity of *Synechococcus* strains (picocyanobacteria) isolated from the Baltic Sea. ISME J. 3:397–408.

**Hayes PK, Barker GLA. 1997.** Genetic diversity within Baltic Sea populations of *Nodularia* (cyanobacteria) 1. J. Phycol. 33:919–923.

**Hällfors G & Hällfors S. 1992.** The Tvärminne collection of algal cultures. In: Tvärminne studies, Zoological Station, University of Helsinki, pp. 15–17.

**Iteman I, Rippka R, Tandeau de Marsac N, Herdman M. 2002.** rDNA analyses of planktonic heterocystous cyanobacteria, including members of the genera *Anabaenopsis* and *Cyanospira*. Microbiology 148:481–496.

**Kononen K. 1993.** Evaluation of the nutrient control hypothesis of the filamentous cyanobacterial blooms in the nutrient recycling ecosystem in the central Gulf of Finland, Baltic Sea. Finn. Mar. Res. 261:1–36.

**Koskenniemi K, Lyra C, Rajaniemi-Wacklin P, Jokela J, Sivonen K. 2007.** Quantitative real-time PCR detection of toxic *Nodularia* cyanobacteria in the Baltic Sea. Appl. Environ. Microbiol. 73:2173–2179.

**Laamanen MJ, Gugger MF, Lehtimäki JM, Haukka K, Sivonen K. 2001.** Diversity of toxic and nontoxic *Nodularia* isolates (cyanobacteria) and filaments from the Baltic Sea. Appl. Environ. Microbiol. 67:4638–4647.

**Lehtimäki J, Lyra C, Suomalainen S, Sundman P, Rouhiainen L, Paulin L, Salkinoja-Salonen M, Sivonen K. 2000.** Characterization of *Nodularia* strains, cyanobacteria from brackish waters, by genotypic and phenotypic methods. Int. J. Syst. Evol. Microbiol. 50:1043–1053.

**Lyra C, Laamanen M, Lehtimäki JM, Surakka A, Sivonen K. 2005.** Benthic cyanobacteria of the genus *Nodularia* are non-toxic, without gas vacuoles, able to glide and genetically more diverse than planktonic *Nodularia*. Int. J. Syst. Evol. Microbiol. 55:555–568.

**Martin C, Sivonen K, Matern U, Dierstein R, Weckesser J. 1990.** Rapid purification of the peptide toxins microcystin-LR and nodularin. FEMS Microbiol. Lett. 56:1–5.

**Moffitt MC, Neilan BA. 2001.** On the presence of peptide synthetase and polyketide synthase genes in the cyanobacterial genus *Nodularia*. FEMS Microbiol. Lett. 196:207–214.

**Sivonen K, Kononen K, Carmichael WW, Dahlem AM, Rinehart KL, Kiviranta J, Niemela SI. 1989a.** Occurrence of the hepatotoxic cyanobacterium *Nodularia spumigena* in the Baltic Sea and structure of the toxin. Appl. Environ. Microbiol. 55:1990–1995.

**Sivonen K, Kononen K, Esala A-L, Niemela SI. 1989b.** Toxicity and isolation of the cyanobacterium *Nodularia spumigena* from the southern Baltic Sea in 1986. Hydrobiologia 185:3–8.

**Yoshida T, Hairston NG & and Ellner SP. 2004.** Evolutionary trade-off between defence against grazing and competitive ability in a simple unicellular alga, *Chlorella vulgaris*. Proc. R. Soc. B 271:1947–1953.
